# Practicing community-based research in GIScience and geography – a case study with an Indigenous community, best practices, challenges, and lessons learned

**DOI:** 10.1080/15230406.2025.2540843

**Published:** 2025-08-28

**Authors:** Yan Lin, Daniel Beene, Theodros Woldeyohannes, Zhuoming Liu, William Tatman, Andee Lister, Xi Gong, Miriam Gay-Antaki, Jani Ingram, Joseph Hoover

**Affiliations:** a Department of Geography, The Pennsylvania State University, University Park, PA, USA;; b Department of Epidemiology, Bloomberg School of Public Health, Johns Hopkins University, Baltimore, MD, USA;; c Department of Geography and Environmental Studies, University of New Mexico, Albuquerque, NM, USA;; d Department of Environmental Science, University of Arizona, Tucson, AZ, USA;; e Department of Biobehavioral Health and Institute for Computational and Data Sciences (ICDS), The Pennsylvania State University, University Park, PA, USA;; f Department of Chemistry and Biochemistry, Northern Arizona University, Flagstaff, AZ, USA

**Keywords:** Community-based research, participatory GIS, indigenous population, environmental health, community geography

## Abstract

Community-based research (CBR) in geography is increasingly emphasizing participatory approaches that center the voices of local communities in the research process. This shift seeks to move away from extractive research practices by fostering collaborations built on reciprocity and respect – particularly with Indigenous and marginalized groups. At the core of this approach is co-produced knowledge, wherein communities actively shape research priorities, methodologies, and interpretations. Rather than imposing external frameworks, these collaborations recognize the value of local and Indigenous knowledge systems in informing research and driving meaningful outcomes. In this paper, we review contemporary CBR literature in geography and GIScience and present a case study on environmental health concerns related to mining legacies in the U.S. This research, led by GIScience and geospatial experts in collaboration with a Tribal community, illustrates opportunities to advance CBR theory and practice within these fields. As CBR becomes increasingly integrated into GIScience projects, we critically examine the positionality of GIScience researchers in this transition, the challenges they face, and the lessons learned. The paper closes with a discussion of best practices for CBR. While all research involves some degree of extractivism, we explore how CBR can help ensure that communities derive direct and tangible benefits from participation in GIScience and geography research.

## Introduction

Community-based research (CBR) implemented within a geography context integrates various methodological frameworks, including Participatory Action Research (PAR), Community-Based Participatory Research (CBPR), Participatory GIS (PGIS), and Indigenous and decolonial methodologies. Previous work has emphasized the importance of reciprocal research partnerships with Indigenous communities, advocating for CBPR as a means to generate research that is both academically rigorous and directly beneficial to those involved (Tobias et al., 2013). Other works highlight both the challenges and opportunities of CBPR, noting that while it fosters meaningful outcomes, it also requires sustained commitment to equity, transparency, and dialogue (De Leeuw et al., 2012; Koster et al., 2012). We review several geographic frameworks that align with CBR, emphasizing research with Tribal communities.

### Community-based research frameworks

Geographers are adopting pragmatism and “geography as praxis” as guiding epistemological frameworks (Shannon et al., 2021). This perspective underscores the need for research that is participatory, action-oriented, and grounded in the realities of the communities it seeks to serve. Pragmatism in geography prioritizes actionable research that addresses real-world challenges, though the emphasis of how this is achieved varies by framework. PAR, for example, integrates research with direct community engagement, allowing participants to play an active role in shaping the research process (Baum et al., 2006). This iterative approach combines cycles of action and reflection to produce knowledge that is not only academically valuable but also contributes to social change (Baum et al., 2006; Cornish et al., 2023). Fischer et al. (2022) used PAR to address social justice concerns while integrating local expertise (Fischer et al., 2022). In another study, Kim (2018) leveraged participatory frameworks to foster co-production; and argued that PAR represents a research philosophy that reshapes power dynamics between academic researchers and community stakeholders (Kim, 2018). A similar work underscores the significance of care and relationship-building in PAR, suggesting that the research process itself is just as crucial as its outcomes (Mason, 2015). These approaches highlight the necessity of ethical engagement, challenging researchers to remain conscious of power dynamics and actively work to mitigate them. Harney et al. (2016) describes “process pragmatism” in human geography, emphasizing continuous reflection on the researcher’s role and its impact on the community (Harney et al., 2016). This ethical framework aligns with Indigenous research principles, as articulated in another work, arguing that cross-cultural geographic research must respect Indigenous sovereignty and prioritize community goals over purely academic objectives (Hodge & Lester, 2006). Building healthy collaborative relationships with Indigenous communities may further be hindered by the traditional structure of academia and granting agencies, which can mandate asymmetrical power over the control of data and knowledge, require publication, and pigeonhole knowledge into specific disciplinary contexts (Coleman et al., 2020). If this distrust can be overcome, there is an opportunity to use mapping within a CBR context to foster empowerment by supporting communities as they represent their spatial realities actively (Jung, 2018). Other notable examples illustrate the various contexts in which GIS is integrated with community engagement to support sustainable redevelopment (Dionisio et al., 2016), and equitable rebuilding project (Duval-Diop et al., 2010).

### Indigenous geographies

Advancing CBR in geography is also increasingly informed by indigenous methodologies, which center Indigenous knowledge systems and emphasize research practices that respect Indigenous sovereignty and self-determination (Daigle, 2016, 2025; Radcliffe, 2022). For geographers who wish to move beyond Western-centric frameworks, Indigenous methodologies reshape CBR by requiring researchers to relinquish control over research agendas, processes, and interpretations, and instead follow Indigenous protocols that emphasize ethical and reciprocal research (Frantz & Howitt, 2012; Tobias et al., 2013). Early critiques of using GIS with Indigenous communities warned that bringing in Western mapping tools without care can push aside Indigenous ways of understanding the land, weaken oral traditions, and replace them with Western modes of thinking (Rundstrom, 1995). Frantz and Howitt (2012) call for a shift toward “conducting research with and for Indigenous peoples,” urging geographers to critically examine colonial legacies, reframe power dynamics, and center Indigenous leadership and priorities throughout the research process (Frantz & Howitt, 2012). This shift aligns with broader efforts to decolonize research methodologies by challenging conventional, extractive models of knowledge production while advocating for research practices that center Indigenous voices, honor relational accountability, and promote sustained, non-exploitative partnerships throughout the research process (Koster et al., 2012). Another study extended this argument by encouraging a post-colonial approach to Indigenous geography that puts Indigenous people in charge of research about their own lives. Instead of relying on outsiders to lead the work, they call for research that supports Indigenous control, protects land rights, and values care for the environment. Their work pushes back against extractive research and calls for more respectful, meaningful partnerships grounded in Indigenous priorities and ways of knowing (Coombes et al., 2014).

However, folding Indigenous voices and identities into research agendas is not straightforward, especially in the context of resource extraction. Valuing Indigenous perspectives means recognizing that the wants and needs of Indigenous groups do not always align with outsider expectations of resistance or environmentalism. As Curley (2018, 2021a) discusses, economies surrounding resource extraction complicate idealized western narratives of Indigeneity and Tribal self-determination, which are not inherently anti-capitalist or limited to traditional ecological knowledge. Indigenous ideology – rooted in place-based understandings, survival strategies, and social obligations – can shape how communities navigate and negotiate capitalist systems. For instance, the willingness or even enthusiasm of some Indigenous peoples to engage in mining reflects more than assimilation; it represents culturally grounded responses to the wage economy imposed by settler colonial states (Eichstaedt, 1994; Pasternak, 2010; Voyles, 2015). Curley (2018) examines this complexity through the moral economy of Navajo coal workers, who frame their labor through the Navajo concept of t’a ahwoajı t’eego – an ethic of self-reliance and hard work tied to traditional land. This understanding is shaped by collective experiences, such as union participation, and serves to mobilize support for a declining coal industry. Curley further reminds us that capitalism and colonialism are entangled with Indigenous sovereignty (Curley, 2021a). As such, decolonial research must engage these lived contradictions – understanding and negotiating them in collaboration with Indigenous partners – rather than smoothing them over to fit outsider ideals.

### Geospatial technologies and methods in CBR

GIS has become a widely used tool in CBR, allowing geographers and communities to integrate spatial data with participatory methodologies to address pressing social and environmental issues. Key areas of application include PGIS, public health, environmental justice, disaster resilience, and Indigenous and counter mapping. PGIS, in particular, has empowered marginalized communities by involving them in mapping and decision-making processes (Dunn, 2007; Elwood & Leitner, 1998; King, 2002). The strength of PGIS lies in its ability to blend local knowledge with scientific data, producing outcomes that are both community-driven and analytically robust (King, 2002). PGIS examples include influencing natural resource management decisions (King, 2002), and GIS-based CBPR initiatives that incorporate diverse neighborhood perspectives into urban planning to address community-specific concerns (Elwood & Leitner, 1998).

However, PGIS has been criticized as to whether it genuinely serves community needs or if it risks reinforcing a “post-political” agenda, in which technical solutions obscure deeper socio-political issues (Dunn, 2007). Recent scholarship suggests that PGIS must move beyond technocratic applications to engage meaningfully with power dynamics and community priorities (Radil & Anderson, 2019). These challenges are broadly applicable to community-based research in geography and other fields. There remain significant barriers to successful participatory research with Indigenous communities including historical and multigenerational mistrust of academics who rely extensively on tools and techniques framed through a positivist, Western world view.

GIS has also played a role in preserving and amplifying Indigenous knowledge through mapping and community engagement. The concept of “process toponymy,” was introduced using GIS to document Indigenous Evenki place-naming systems in an open-access platform (Mamontova & Klyachko, 2022). The integration of GIS with vernacular cartography has also proven effective in preserving Indigenous place names and traditional ecological knowledge, ensuring that local histories and cultural identities are maintained in digital mapping platforms (Mamontova & Klyachko, 2022). Another work examined how mine closure plans in Northern Canada have (or have not) incorporated Indigenous knowledge, highlighting the need for more inclusive and participatory planning processes (Monosky & Keeling, 2021). The value of resiliency-stressor frameworks was emphasized in environmental justice mapping, supporting residents of Indigenous communities as they develop long-term solutions (Burwell-Naney et al., 2019). Advancement in decolonial methodologies include “walking as mapping” in Mixteco geographies, demonstrating how embodied spatial practices contribute to geographic knowledge production in meaningful ways (Alcantar, 2024). Collectively, these studies underscore the potential of GIS, when combined with Indigenous methodologies, as both a tool for spatial data representation and a means of fostering self-determination, empowering communities to lead their own spatial research and decision-making processes. This perspective aligns with our research by emphasizing the need to center Indigenous voices and knowledge systems in spatial analysis and GIS.

### Opportunities to grow community geography

Building on decolonial and Indigenous methodologies, community geography extends beyond participatory research by critically engaging with power dynamics within the research process (Fischer et al., 2022; Shannon et al., 2021). The intersectional dimensions of gender and climate change have been highlighted in geographical research emphasizing that addressing power differentials is crucial for producing truly inclusive and representative outcomes (Sultana, 2014). Similarly, “boundary work” has emerged as an innovative approach within community-based and Indigenous geography, aiming to bridge academic and Indigenous knowledge systems to support collaborative efforts on food sovereignty and environmental justice (Zurba et al., 2019). In practice, boundary work involves the co-creation of “boundary objects” – tools such as maps, models, or shared frameworks – that help communicate knowledge, values, and aspirations across social, cultural, and political divides. By facilitating mutual understanding and dialogue, boundary work enables researchers and Indigenous communities to collaboratively define research goals, practices, and outcomes. It repositions Indigenous voices at the center of the research process, aligning efforts with community priorities and reinforcing principles of self-determination and respect for Indigenous ways of knowing.

Efforts to formalize community geography have gained momentum, aiming to establish disciplinary frameworks that guide participatory research. A comprehensive overview of “doing community geography” emphasizes co-produced knowledge and the balance between academic rigor and community relevance through mutually beneficial partnerships. Shannon et al. (2021) proposed a community geography framework that addresses practical challenges such as sustaining long-term community partnerships, equitably sharing data, and navigating funding constraints (Shannon et al., 2021). Other studies contribute to this discourse by evaluating public participation in geographic research, highlighting the role of educational institutions in training the next generation of community-based scholars (Hawthorne & Jarrett, 2018; Robinson, 2010).

A community geography framework also calls for decolonizing research to dismantle the legacy of colonialism in academic geography (Nelson & McGregor, 2014). Decolonizing efforts advocate for shifting from research “on” to research “with” communities, ensuring collaborative practices lead to empowering outcomes (De Leeuw et al., 2012). Recent scholarship has expanded the framework to emphasize transdisciplinary and participatory innovations, particularly through GIS, participatory mapping, and citizen science to address environmental justice and resilience (Haklay & Francis, 2017; Kumasaka et al., 2022). These innovations integrate Indigenous and scientific knowledge to tackle challenges such as land reclamation, self-determination, and environmental health.

Despite progress, several gaps remain in how geographers engage with communities, particularly regarding ethical research practices in collaborations with marginalized and Indigenous groups. First, persistent concerns exist about power imbalances between researchers and communities, especially when research is driven by academic goals rather than community priorities. Scholars advocate for non-extractive research ethics, calling for frameworks that respect Indigenous epistemologies and challenge traditional power imbalances (Kouritzin & Nakagawa, 2018). This aligns with previous work emphasizing producing respectful and reciprocal research through CBPR with Indigenous populations (Tobias et al., 2013). Research further argues for non-extractive methods that prioritize community benefits over academic recognition (Igwe et al., 2022; Koster et al., 2012).

Second, while Indigenous geographies have gained traction, challenges remain in integrating these epistemologies and methodological approaches within academic frameworks. The dominance of Western epistemologies often marginalizes Indigenous approaches, necessitating deeper engagement with Indigenous ways of knowing (Frantz & Howitt, 2012; Hodge & Lester, 2006). There is a pressing need for methodological diversity, particularly in integrating Indigenous sciences with Western scientific frameworks (Kater, 2022). This includes participatory approaches that extend beyond data collection to incorporate storytelling, Indigenous knowledge, and other culturally significant methods.

Finally, sustaining long-term community engagement remains a challenge, as funding constraints and institutional barriers often limit the duration of partnerships. A study explored the precarity of researchers engaged in community geography, highlighting the difficulties of maintaining mutually beneficial relationships within academia’s demands for measurable outcomes (Barrett & Bosse, 2022). Additionally, a lack of robust metrics for evaluating the impact and effectiveness of participatory approaches persists. Another study stressed the importance of frameworks that assess both community empowerment and academic outcomes (McCall & Minang, 2005). Recognizing the significance of training future community-based scholars, another study discussed the need for mentoring young geographers in community-engaged research, yet capacity-building efforts remain inconsistent across institutions (Hawthorne & Jarrett, 2018).

Literature on community-based geography reflects a field in transition, moving from traditional research paradigms to more inclusive, participatory, and ethically grounded approaches. While significant progress has been made in developing frameworks and methodologies that prioritize community needs and empower marginalized voices, challenges related to power dynamics, funding, and methodological adaptation remain. We present a case study addressing geospatial patterns of contaminant exposure in a Tribal community to highlight opportunities to apply current CBR theory and methods.

## Case study: addressing community environmental health concerns through geography

**Figure 1.**
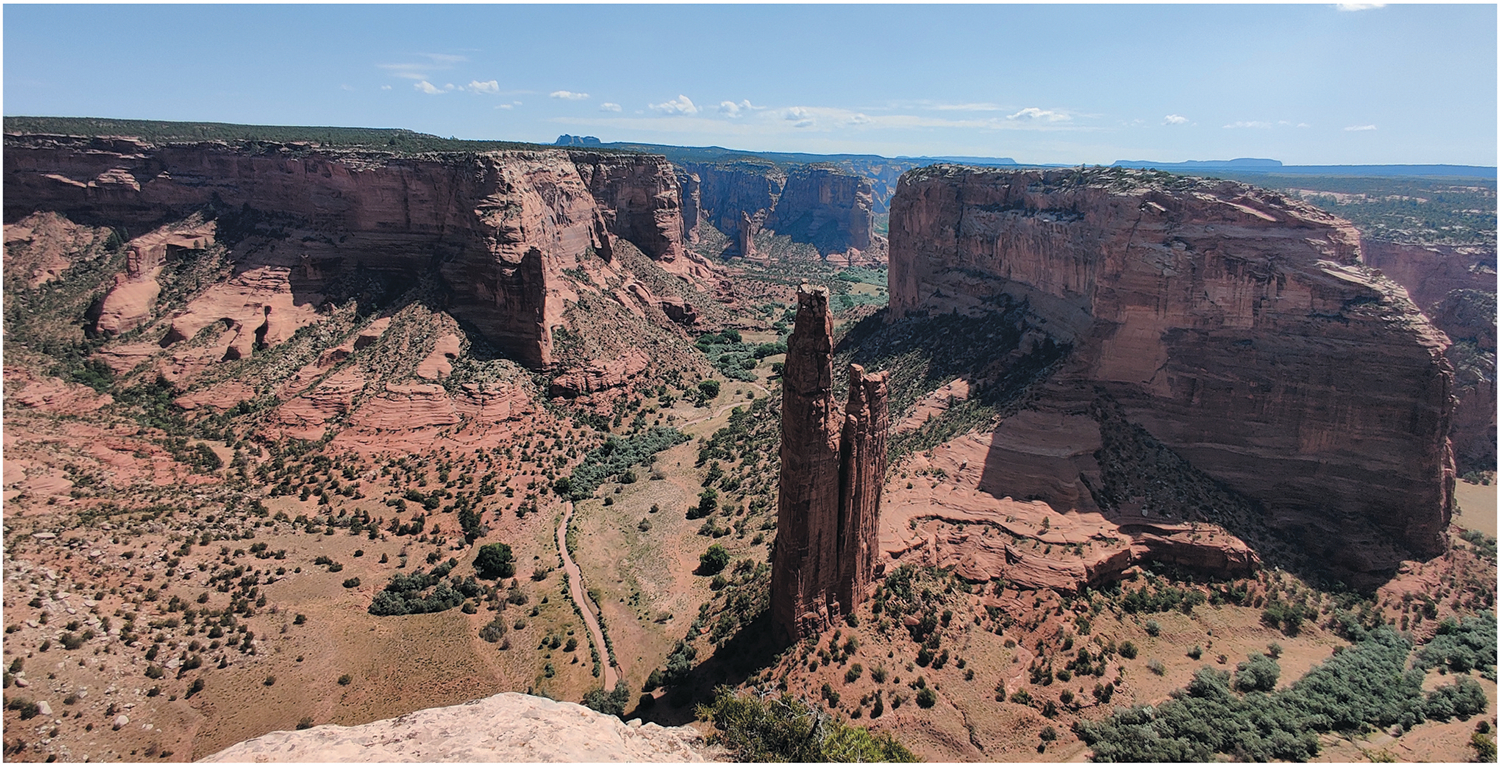
Canyon de Chelly National Monument as viewed toward Spider rock.

The Navajo Nation is the largest Tribal reservation in the US covering roughly 70,000 square kilometers in the southwestern U.S. During a recent community trip, our team passed Spider Rock at Canyon de Chelly National Monument (Figure 1). We stood in this sacred canyon – home to Diné families who have long raised livestock, cultivated the land, and sustained life – their enduring presence woven into the landscape. These homes and vistas, etched with their stories, resonate deeply, reminding us to honor this place by contributing to the restoration of its peace, wellbeing, and harmony. As community geographers, spaces such as Spider Rock remind us of the critical importance of centering the community, Indigenous epistemologies, values, and lived experiences in our research process.

In this case study, we present a summary of work conducted by a transdisciplinary research team working alongside Indigenous communities to address concerns about the legacy of hard rock mining on livestock health. We present this work in the context of federal settlements on uranium mine cleanup and seek to highlight examples of co-produced research that uplifts Indigenous knowledges and cultural practices and is informed by positivist research techniques from GIScience and environmental chemistry. Our decision-making process was guided by a community based participatory research (CBPR) framework to foster project co-development, implementation, training, and dissemination of results in a manner appropriate for community members and leaders.

### Historical context of hard rock mining

**Figure 2.**
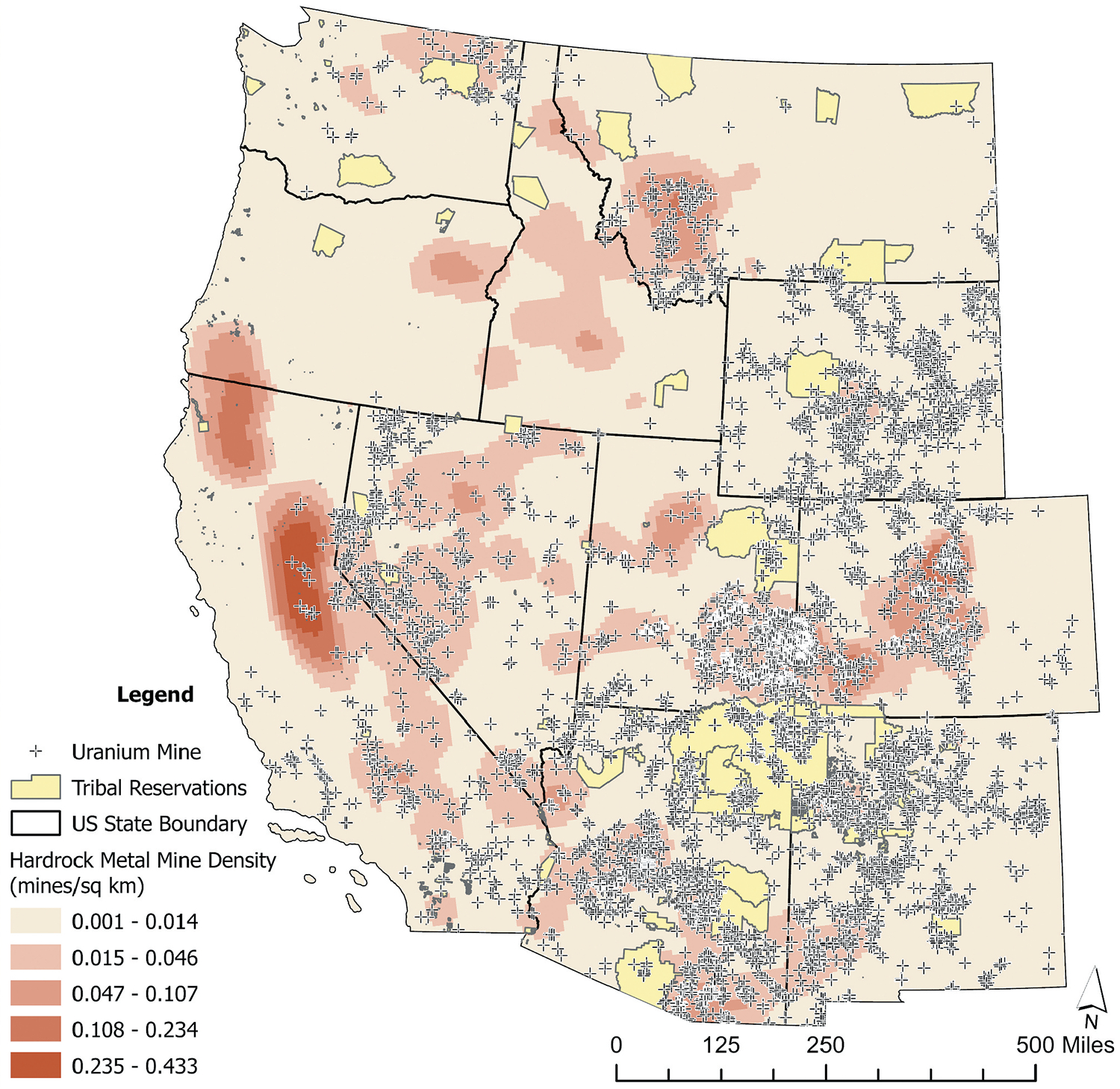
Hard rock mine density across the western United States (Note: reservation land is indicated by yellow polygon areas, mine densities are associated with intensity of red hues, and abandoned uranium mine sites are indicated by the crosses.).

This case study was developed in response to decades of community concerns regarding exposure to mine wastes from hard rock mines throughout the Western U.S. The presence of more than 160,000 hard rock mines has created a legacy of chronic exposure to metal mixtures among Indigenous communities (Lewis et al., 2017). (Figure 2) illustrates the density of hard rock metal mines in the Western U.S., including abandoned uranium mines (AUMs), a type of hard rock mine of particular importance for residents of the Navajo Nation and other Indigenous communities. It was reported that approximately one in five AUMs are located within 10 km of a Reservation (Lewis et al., 2017).

Uranium mining in the Navajo Nation lasted from 1944 to 1986 during the Cold War, producing about 30 million tons of uranium for nuclear development. Of the more than 4,000 AUMs in the Western U.S., approximately 500 are located in the Navajo Nation. While occupational exposure and lung cancer among uranium miners are well-documented, there is more limited, though growing, research on the association between AUM exposure and health outcomes. Previous research has documented that Indigenous Peoples who practice land-based cultures are intimately connected to the environment due to cultural, spiritual, and traditional practices, which may result in different magnitudes of exposure when compared to other populations in the United States (Fegadel, 2023). Existing biomedical and environmental exposure studies have found that AUM exposure is linked to increased risks of cardiovascular disease, higher antibody levels among older populations, preterm birth, and other negative health outcomes (Erdei et al., 2023; Hoover et al., 2023; Lewis et al., 2017).

Indigenous communities have played a crucial role in addressing legacy and on-going challenges to mining impacts on their people, communities, and lands. Communities and labor organizations identified problems, organized to learn about them, and formed alliances to address them (Brugge & Goble, 2002). After decades of effort by the Navajo people, the federal government launched a coordinated effort to address uranium contamination on the Navajo Nation in 2008. Subsequently, the U.S. Environmental Protection Agency (EPA) entered into enforcement agreements and settlements with the Navajo Nation and mining companies to address contaminant challenges, totaling approximately $1.7 billion for risk reduction measures planned for 230 AUMs. These funds come from former mining companies and are administered by the US EPA in consultation with the Navajo Nation. However, the estimated cost for cleanup and risk reduction far exceeds the settled amount; it is anticipated that the $1.7 billion will address only about 10% of abandoned uranium mine waste found across the Navajo Nation. In 2014, the United States reached a historic legal settlement that allocated nearly $1 billion to investigate and clean up approximately 50 uranium mines on or near the Navajo Nation that had been operated by Kerr-McGee Corporation and its successor, Tronox. One community located in the Northern Agency of Navajo Nation in northeastern Arizona hosts 32 of these Tronox-settlement mines. Investigations into the extent of mine impacts commenced as soon as settlement funds were received in January 2015.

Although some work has been conducted on water, soil, plants, and crops in this area of the Navajo Nation (Mares, 2022), livestock, more specifically sheep and goats, have been inadequately integrated in previous environmental evaluations and health risk assessments, deviating from community and cultural norms (Rock & Ingram, 2020). Sheep ownership holds deep cultural and economic significance within Indigenous communities, particularly among the Diné (Navajo) people. Additionally, sheep ownership is shaped by generations of traditional knowledge, representing a nexus of cultural identity, ecological stewardship, and sustainable living. Traditional sheep consumption on cultural rituals, food traditions, and communal gatherings underscores sheep’s role as a traditional and cultural food staple (Rock et al., 2019). Moreover, there remains opportunity for collaborative research partnerships with Indigenous communities to ensure that livestock practices support community health and food sovereignty, especially given the challenges of environmental contamination in groundwater and land (Credo & Ingram, 2021).

The need for risk assessments incorporating Indigenous knowledge is an important start to address the holistic needs of Navajo residents (Rock & Ingram, 2020; Samuel-Nakamura, 2020). Livestock has been overlooked in human health risk assessments, instead, the approach of avoiding contamination at all costs is preached. For example, exposure to uranium in the natural environment occurs primarily through eating contaminated food or drinking contaminated water. The EPA has yet to set a Maximum Contaminant Level (MCL) for uranium in food (Roberts, 2012). Livestock, such as sheep, may roam open areas of a watershed and may come into contact with these unmarked, unfenced AUMs. This provides an opportunity for livestock animals to uptake uranium through numerous pathways. Members of the partner community voiced their concerns about potential environmental uranium contamination in their livestock and raised questions about the safety of consuming livestock from this area. As a team of community-based researchers, we perceive this narrative to reflect a community demanding a greater role in scientific research and decision-making processes impacting their lives (Corburn, 2007). Given these lived experiences and cultural sensitivities, we approached this study with caution and respect.

### Foundation - community partnership

The study team has established and maintained long-standing, trust-based partnerships with multiple Indigenous communities. Our team’s expertise lies at the intersections of environmental health, exposure science, environmental epidemiology, geology, geography, chemistry, and environmental science. The team included experienced Indigenous researchers and trainees (i.e. undergraduate and graduate students as well as postdoctoral fellows) engaged in mentored research with Indigenous communities.

Building on a wide-ranging set of lived experiences and academic disciplines, our team developed research infrastructure that facilitates the design and implementation of research projects centered upon community-driven questions and concerns (Erdei et al., 2023; Hoover et al., 2023; Lewis et al., 2017; Lin et al., 2020; Lister et al., 2023, 2024). This includes training and tools for data acquisition, analysis, and interpretation critically for developing community-driven insights. Equally important, we rely on bi-directional communication between academic-based team members and community-based team members to design and implement projects, which provides meaningful opportunity for research findings that are co-produced, fostering sustained engagement and collaborative knowledge creation.

### Collaboration plan

In the literature, scholars have emphasized the importance of developing a detailed collaboration plan that outlines the overall goals and vision, team composition and responsibilities, collaboration readiness, strategies for community report-back, conflict resolution, communication mechanisms, team expectations, and required training before project implementation (Hall et al., 2014). While the benefits of such plans are well documented, challenges often arise in translating them into practice within community settings. Our team recognized that the communities where we worked have a governance structure that facilitates oversight of projects such as this livestock and environmental contamination study. Therefore, we worked with project partners and the community leadership to craft a resolution of support that articulated clearly the project partners, key roles, data ownership, and engagement/report back expectations. This resolution formed the foundation of all future interactions with the community and its residents through the project.

### Vision and goals

The vision of this collaborative effort was to address community concerns related to AUMs and livestock exposure through enhancing understanding of cumulative exposure pathways, which ultimately support decision-making.

### Key projects and anticipated outcomes

Key project objectives include: determine the frequency and duration of livestock grazing in proximity to abandoned mines and waste; determine cumulative environmental exposure to the livestock; compare uranium levels in tissues and organs from livestock grazing in mining versus non-mining areas; compare uranium levels in environmental samples from grazing areas in mining versus non-mining.

### Training and capacity building

Community members, undergraduate and graduate students, and Indigenous trainees participated in hands-on training in GIS, GPS collaring, field sampling, and basic environmental health research practices. Training materials, including flyers, protocols, and manuals – were co-developed with community partners and tailored for local use.

### Community report-back

Regular community report-backs were a cornerstone of the collaboration plan. The team hosted multiple community meetings – both at project initiation and following major research milestones – to provide updates, discuss findings, and co-interpret results. Results were also shared directly with individual livestock owners in formats that emphasized transparency and accessibility, such as maps, simplified visuals, and flyers.

### Study team and coordination

The study team included community members, local leadership, and research partners from Diné College and Northern Arizona University (NAU). The team was collectively responsible for identifying research questions, designing the study, planning and executing participant recruitment, developing data collection protocols, implementing action plans, analyzing and interpreting data, translating and disseminating findings, managing resources, supporting evaluation efforts, serving on hiring committees, overseeing compliance with Institutional Animal Care and Use Committee (IACUC) approvals, and maintaining communication with extended team members, partners, and other stakeholders. For example, a co-designed recruitment flyer through a iterative process was used for the study which outlined the following components: (1) how the study works: tracking grazing patterns, collecting and testing animal organs for heavy metals; (2) what participation involves: use of GPS collars, organ sampling, and incentives; (3) eligibility: livestock owners in the study area willing to participate; and (4) contact information. Most importantly, such flyers emphasized community involvement and traditional lifeways while inviting participation in an important environmental health study.

The full team met virtually at least once a month to ensure open communication, provide updates, make decisions impacting the research and report back process, and adjust project objectives as needed. Approval of any sub-project, activity, or method of reporting/dissemination required consensus from the team. Component leads (e.g. geospatial analysis, community engagement, and environmental chemistry) meet weekly with their respective teams to facilitate project progress. Team leaders were responsible for fostering a shared understanding of the project among all participants, including students and research partners. This role encompassed maintaining collaboration and mutual respect, ensuring that all team members adhered to these principles while engaging in open and regular communication. Within this collaborative framework, the study was co-designed to assess the frequency and duration of livestock grazing near abandoned uranium mines and waste sites, as well as to evaluate cumulative environmental exposure in livestock through collection and analysis of environmental samples (i.e. soil, water, plants) and tissue from sheep and goats.

### Data governance and ethics

Data governance was guided by the principles of Indigenous data sovereignty and the Navajo Nation’s research oversight framework. The resolution of support clarified that data collected through the project belonged to the community and that any use or dissemination beyond the local context would require approval. IACUC protocols were followed, and additional measures were taken to ensure culturally sensitive data collection and interpretation.

### Timeline

The project unfolded over multiple phases since 2018, with community engagement and trust-building preceding field data collection. Phase 1 involved initial livestock tracking and sample analysis before the pandemic, followed by expanded data collection in Phase 2.

### Community engaged data collection, analysis, and discussion

#### Tracking livestock movement

**Figure 3.**
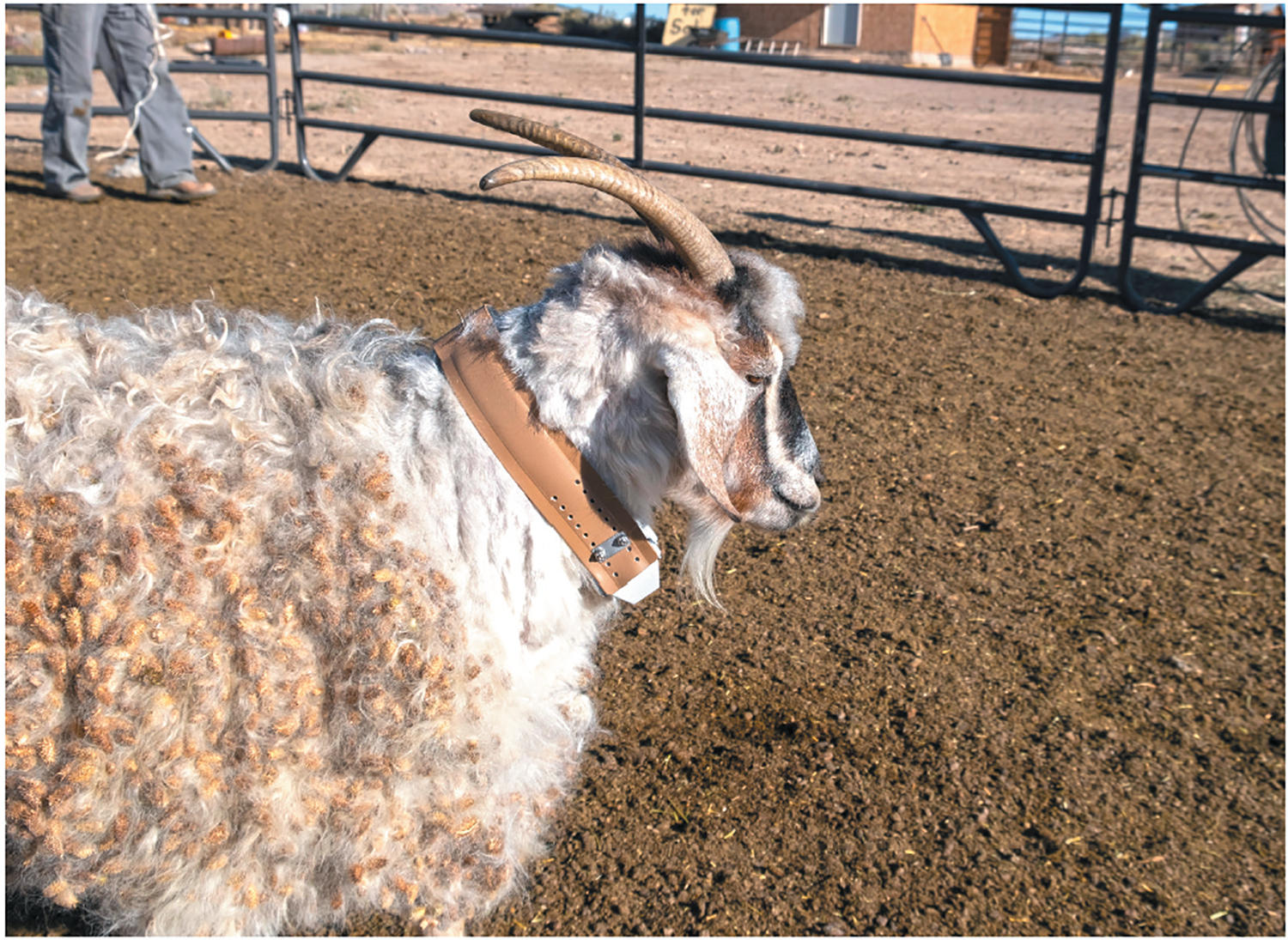
Goat wearing Lotek GPS collar.

To assess livestock movement patterns, we proposed several tracking methods based on the literature, including daily logs, camera/video surveillance, and GPS tracking. After discussions with livestock owners and community members, GPS collars were selected as the preferred method for data collection. We used Lotek GPS collars (Figure 3), which are lightweight and have a battery life exceeding 12 months. With the guidance of community members, we identified representative grazing areas for data collection. Our data sheets recorded livestock food and water sources, grazing patterns, and any additional observations shared by the community.

**Figure 4.**
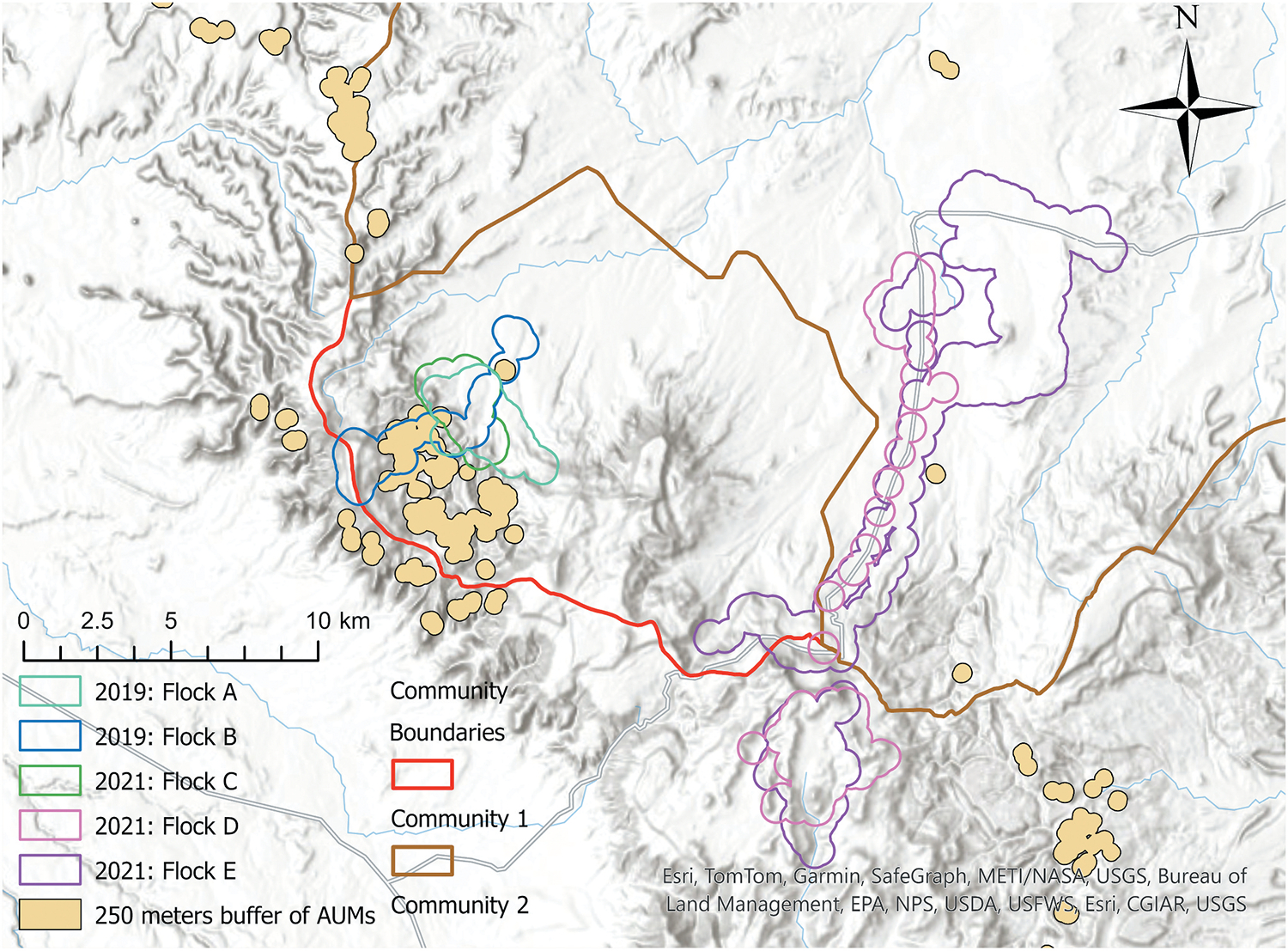
Grazing locations of flocks in the study area encompassing two communities.

GPS tracking was conducted in 2019 and 2021, with a pause in 2020 due to the COVID-19 pandemic. We tracked 21 animals from six flocks, collecting over 100,000 GPS points. After postprocessing, 84% of the data points were validated for further analysis. (Figure 4) presents the AUM buffer areas and grazing locations of four sheep and goat flocks-three in the community and one in a nearby community without uranium mines. Our findings indicated that most collared livestock did not graze near AUMs, with only Flock B briefly interacting with AUMs while moving between the home corral and a summer camp location at higher elevation. These findings directly addressed a key community question: whether livestock animals had any interaction with AUMs and, if so, how frequently. Further details of the analysis can be found in Liu et al. (2023).

#### Holistic risk assessment and integration of Indigenous knowledges

A holistic approach to risk assessment is essential when working with Indigenous communities, ensuring that Indigenous knowledges are integrated into environmental risk assessment and policy development. Traditional environmental risk assessments primarily rely on proximity to AUMs to evaluate contamination exposure, overlooking the interconnected pathways of environmental contaminants (e.g. water, air, soil, and vegetation) (Rock & Ingram, 2020). Moreover, existing methodologies often fail to account for meteorological and terrain effects, and robust datasets for sophisticated models are often unavailable in rural areas like the Navajo Nation.

Incorporating the lived experiences of local communities and the insights of stakeholders is crucial for conducting comprehensive and effective environmental assessments, as these perspectives ensure that evaluations are grounded in real-world contexts and address the needs and values of those directly affected (Coombes et al., 2014; De Leeuw et al., 2012; Zurba et al., 2019). To address these gaps, we developed a community-based geospatial model through an iterative process to estimate environmental exposure potential to AUMs on the Navajo Nation. This model leveraged existing geospatial data, field data, and community and stakeholder input, guided by Indigenous knowledge.

The selection of data layers was informed by the community members and existing literature. The final geospatial model incorporated multiple environmental layers, including proximity to AUMs, wind index, topographic wind exposure, downslope drainage, roads, groundwater, and vegetation robustness. These layers were integrated using a GIS-based Multi-Criteria Decision Analysis (GIS-MCDA) and a fuzzy Analytic Hierarchy Process (AHP) to generate an exposure potential map. This method was chosen for its simplicity and interpretability, making it accessible for community partners while also enhancing their research capacity.

Among the environmental data layers, wind patterns emerged as a critical factor supported by both scientific literature and community observations. However, concerns arose regarding the accuracy of available wind data due to limited meteorological monitoring on rural reservation lands. To address this, we collaborated with residents and the Navajo Nation EPA to compile all available wind datasets – long-term and short-term field-based meteorological station data on or near the reservation, Meteorological Terminal Air Report (METAR) data from nearby airports through the Automated Surface Observing System (ASOS), modeled grid data from NOAA, and data collected by our team. We then compared the performance of each dataset and their combinations. Our findings indicated that local field-based meteorological station data provided the most accurate modeling results, reinforcing the necessity of expanding local monitoring infrastructure for improved environmental assessments. Further details on this analysis can be found elsewhere (Girlamo et al., 2023; Lin et al., 2020).

#### Livestock exposure assessment and spatial-temporal modeling

For livestock exposure assessment, we collected over 100,000 GPS points and integrated livestock owner input, including knowledge of animal routines and behaviors to enhance and validate geospatial analysis results. Using fuzzy logic, we accounted for behavioral uncertainties and developed a spatial-temporal model to estimate cumulative exposure potential. This model leveraged the previously developed environmental risk assessment layers, calculating risk for various behavioral scenarios and deriving a daily cumulative exposure potential. To improve computational efficiency, we reduced the number of GPS points and leveraged advanced computing resources for parallel processing. Our methods, detailed in Liu et al. (2023), demonstrated that incorporating livestock behaviors and local knowledge produced results that are closely aligned with community-reported grazing patterns.

#### Tissue analysis and linking to exposure metrics

To address the final research question regarding uranium concentration in livestock, our research partners measured uranium concentrations in muscle, kidney, and liver tissue from sheep and goats (Lister, 2024; Lister et al., 2023, 2024). Preliminary results suggest uranium concentrations were higher in tissues and some organs (e.g., kidney) of sheep that grazed in the community in comparison to sheep grazing in non-mining reference sites (Lister, 2024; Lister et al., 2023, 2024). Further research on sheep consumption suggests that Navajo people may prefer to consume muscle tissue over organ tissue (Nez et al., 2020). The daily consumption of Navajo households (e.g., average sheep consumption per day) is easy to quantify based on past work; however, the lack of a sheep consumption rate from regulatory agencies has not been established, thus the tissue/organ consumption for all Navajo people is unknown (Nez et al., 2020). However, the small sample size limits the generalizability of these findings.

When we linked the modeled cumulative exposure results with measured livestock uranium and arsenic concentrations, we found a weak but statistically significant positive correlation. However, the small sample size restricted the ability to establish stronger statistical significance or broader applicability.

#### Engagement and report back

Throughout the project, our team organized meetings to receive community feedback (e.g. on tracking method selection), discussed and addressed resident concerns, and enabled adaptive modifications to study implementation and objectives. Once underway, team members conducted results report back discussions with the goal of facilitating dialogue about the project and findings. The multi-institutional team created tools and presentation formats that align with community preferences, including fliers, and community report-back meetings. We maintained frequent communication with individual livestock owners, particularly to share results such as maps depicting livestock movement patterns and proximity to AUMs. Livestock owners provided feedback to discuss, and ultimately validate, map accuracy and suggested additional research questions to explore. For example, in efforts to classify animal behaviors using computer algorithms, livestock owners contributed detailed knowledge about typical grazing, traveling, and resting patterns – enabling more accurate interpretation of GPS data. Their input was essential not only for improving classification accuracy but also for validating the resulting behavioral categories. One analytical challenge faced by the geospatial team was the need to reduce the number of GPS points due to computational limitations and the NP-hard nature of certain spatial problems. Livestock owners addressed this issue by identifying periods when animals remained in corrals or were inactive, which allowed the research team to eliminate unnecessary data points and enhance the efficiency of the analysis. Several community meetings were held to introduce key team members, present preliminary study plans, and obtain community approval through resolutions of support, congruent with the community governance structure. These meetings created space for community members to voice concerns and ideas, fostering a participatory study design. Two community report-back meetings were held to present the study findings and receive feedback on the research process. Study results were first disseminated at the community level – starting with individual livestock owners – before being shared elsewhere, ensuring alignment with community priorities and approval.

While no formal evaluation instrument (e.g. post-meeting surveys or structured interviews) was employed to evaluate the effectiveness of these report back, we gathered informal feedback through open dialogue during and after meetings. Community members expressed appreciation for the opportunity to see visualizations of livestock movement and preliminary data and results. Several participants shared that they felt their voices were heard and reflected in the research process. One livestock owner noted that the detailed maps helped him better understand his animals’ grazing range. Others requested copies of the maps for their own records, underscoring the practical usage of the information. The community’s continued engagement, iterative feedback, and participation in follow-up meetings and planning discussions suggest that the report-back approach was viewed as useful and respectful.

## Challenges, and future opportunities for growing community geography

Upon reviewing collected and analyzed geospatial, environmental, and tissue data, our team concluded that livestock in mining communities were grazing in closer proximity to abandoned uranium mines (AUMs) and that uranium concentrations in livestock were higher in comparison to those in reference to non-mining communities. We acknowledged the potential bias due to the relatively small sample size. These findings are only based on a subset of animals analyzed in Phase 1, as laboratory analysis for samples from Phase 2 is still ongoing. In the report back, livestock owners played an important role in interpreting the preliminary results. For example, one owner explained that his sheep primarily grazed near his home and were supplemented with hay and feed sourced from the nearby Navajo Agricultural Products Industry (NAPI). In contrast, another owner reported that their sheep grazed in canyon areas where runoff from mine waste was known to be present at high concentrations. These contextual insights were critical for understanding and interpreting the observed results in uranium exposure. However, this does not mark the conclusion of Phase 1 research efforts. From a conventional academic standpoint, these findings might be seen as conclusive, suggesting that we move forward to another phase. However, through a lens of community geography, we recognize other key considerations and limitations that suggest a different path forward.

As a community-based study, respecting community decisions and priorities is paramount. In this case, community members raised further questions: How representative were the sampled animals? Where should livestock graze? How can livestock animals be safe from contamination? Is it safe to consume livestock? To address the first question, we acknowledged that the recruited livestock were not fully representative of the entire study area or all grazing patterns; rather, they were based on voluntary participation and not all livestock owners chose to enroll. As discussed earlier, livestock play a central role in the livelihoods, cultural practices, and overall wellbeing of the community. Curley (2021b) argued that livestock can carry deeper cultural and historical meanings that go beyond colonial control and challenge simple economic explanations. However, the study samples may not be representative of all grazing conditions, as they may lack sufficient animals that graze closer to mine sites or consume different food and water sources – such as livestock relying on well water potentially contaminated by AUM waste versus uncontaminated sources, or hay sourced locally versus from outside the region. Furthermore, the COVID-19 pandemic disrupted recruitment efforts, further limiting the scope of our sample.

Many of the remaining questions raised by the community were not easily addressed by the research team. Simply publishing the study, as is typical in academic work, did not meet the needs or priorities of local residents. Instead, data and preliminary findings – specifically the detection of elevated uranium concentrations in livestock from mining areas – were shared directly with the U.S. EPA. This marked the first time uranium contamination in livestock was formally documented and reported, adding a critical new exposure pathway to existing evidence of contamination in water, air, plants, and soil. Despite these findings, significant gaps remain in the scientific literature regarding what constitutes “safe” consumption of meat from livestock exposed to uranium and arsenic. Community members continue to seek guidance on consumption standards, but a lack of data leaves this unresolved issue. Nevertheless, the community has played a central role in bringing visibility to these environmental injustices, supported by ongoing collaboration with researchers, including our team. As a result of these collective efforts, on 5 March 2024, the EPA added the Lukachukai Mountains Mining District – home to the study sites – to the Superfund National Priorities List (NPL) (U.S. Environmental Protection Agency, 2024). This designation acknowledges the serious health and environmental risks from legacy uranium and vanadium mine waste and makes the area eligible for long-term federal cleanup funding under the Superfund program, marking a significant step forward for environmental justice in a region long burdened by contamination. For GIS and geospatial experts, our training focuses on data collection, analysis, and methodological rigor. However, challenges arise in real-world applications, particularly in navigating conflicts among academic research, government policies, and community needs, especially when working within the complex historical and political context of mining legacies. While GIS and geospatial science have traditionally offered technical solutions, community-based research necessitates an expanded skill set that integrates interdisciplinary knowledge, cultural awareness, and an understanding of the broader socio-political landscape. In our case, the research helped build both the scientific and community-based evidence supporting the need for comprehensive federal cleanup in the Lukachukai region. We did not limit our efforts to data collection and analysis, nor did we wait for final results or a perfect publication. Instead, we exposed our research findings to the State to address urgent community concerns and help drive meaningful change.

Leading a community-based project extends beyond data collection, analysis, and reporting. It requires stepping beyond our disciplinary comfort zones to engage meaningfully with the community’s concerns and priorities and experts in other disciplines. A deep understanding of the history, context, and lived realities of the affected communities is foundational for building respectful and authentic relationships. In order to engage respectfully, it is essential that external researchers recognize that community needs are not monolithic; they are shaped by diverse perspectives and priorities and acknowledging this plurality before entering the community or launching a project is crucial.

In our study, the initial framing of environmental harm as the primary concern may not have fully reflected the community’s priorities – particularly given the economic and cultural importance of livestock. Curley’s (2018) moral economy perspective offers a valuable lens to better understand these dynamics. Importantly, such research must be truly community-centered, flexible, and responsive to changing needs, while also acknowledging that “community” is not monolithic. Diverse and, at times, conflicting perspectives exist within communities, and respecting these differences is essential for community-based research. Furthermore, conducting community-based research inherently involves uncertainties – extended timelines, unforeseen barriers, and, at times, the absence of usable data, which may mean that anticipated academic publications do not materialize. In today’s academic environment, this poses challenges for researchers engaged in community-based work.

### Best practices: reflection on our approach and what we learned

Strong and lasting relationships with the community were at the heart of the case study presented here. From the beginning, we worked closely with community members – not just to get approval but to make sure the research truly reflected their questions, needs, and values. This meant involving the community at every step: shaping the research questions, choosing how to collect data, helping analyze what we found, and deciding how to share results. We followed the principles of community-based research and worked hard to respect local knowledge, leadership, and traditions. This approach also came with challenges. Academic and community timelines don’t always match, and it took time to build trust and adjust plans based on community feedback. There were also technical hurdles, like working in rural areas with limited data or tools. But because we stayed in regular contact and made space for open conversations, we were able to adapt.

While we didn’t formally measure how effective the engagement process was, we saw clear signs it made a difference. Community members stayed involved, asked important questions, and helped shape the direction of the study. They also requested follow-up work, and our shared findings directly contributed to a major outcome that the Lukachukai Mountains Mining District added to the NPL. This designation opens the door for long-term federal cleanup and support. It is a powerful example of how community-based research, when done respectfully and collaboratively, can drive meaningful changes.

Overall, our case study showed that doing research “with” a community – not “on” it – requires more than just good methods. It requires trust, flexibility, respect, and a shared commitment to making a difference. We hope this model can help others working in geography and GIS who want to do meaningful, community-centered research.

## Conclusions

CBR in geography has evolved to emphasize participatory methods, prioritizing local voices and shifting away from extractive research practices. Scholars have increasingly adopted frameworks like PAR, CBPR, PGIS, and Indigenous methodologies to foster ethical and reciprocal engagement. These approaches challenge traditional power dynamics by integrating local and Indigenous knowledge systems, ensuring that research is not only academically rigorous but also meaningful to the communities involved. GIS has played a pivotal role in this shift, enabling researchers and community members to collaboratively address pressing social and environmental issues through spatial analysis and mapping. However, challenges persist, including balancing academic and community priorities, navigating institutional constraints, and sustaining long-term partnerships. To address these concerns, geographers must embrace interdisciplinary training, reflexive research practices, and sustained engagement strategies that respect Indigenous sovereignty and community-driven priorities. A clear collaboration plan, outlining research goals, responsibilities, communication strategies, and ethical considerations – can enhance the effectiveness of GIS and geospatial experts in community-based research, ensuring that their work remains inclusive, impactful, and ethically grounded.

## Data Availability

Data cannot be made publicly available due to protecting research participant privacy under the Institutional Animal Care and Use Committee (IACUC).

## Key policy highlights

- community-based research to address community priorities and benefit communities directly
- integrate Indigenous knowledge systems and respect Indigenous sovereignty in data collection, interpretation, and dissemination
- inform policies addressing environmental health risks in Indigenous communities
- community-centered decision-making with transparency and accountability in research
- community capacity building and training to be integrated into research for sustainability

## References

[R1] Alcantar ES (2024). Indian time, walking as mapping, and decolonial methodologies in Mixteco geographies. The Professional Geographer, 77(4), 525–531. 10.1080/00330124.2024.2372823

[R2] Barrett E, & Bosse AJ (2022). Community geography for precarious researchers: Examining the intricacies of mutually beneficial and co-produced knowledge. Geo Journal, 87(s2), 159–170. 10.1007/s10708-020-10358-2 33519040 PMC7835441

[R3] Baum F, MacDougall C, & Smith D (2006). Participatory action research. Journal of Epidemiology and Community Health, 60(10), 854–857. 10.1136/jech.2004.028662 16973531 PMC2566051

[R4] Brugge D, & Goble R (2002). The history of uranium mining and the Navajo people. American Journal of Public Health, 92(9), 1410–1419. 10.2105/AJPH.92.9.1410 12197966 PMC3222290

[R5] Burwell-Naney K, Wilson SM, Whitlock ST, & Puett R (2019). Hybrid resiliency-stressor conceptual framework for informing decision support tools and addressing environmental injustice and health inequities. International Journal of Environmental Research and Public Health, 16 (8), 1466. 10.3390/ijerph16081466 31027209 PMC6518295

[R6] Coleman L, Apok C, Carothers C, Ambrozek S, Zanotti J, & Huang C (2020). Political ecology and decolonial research: Co-production with the Inupiat in Utqiagvik. Journal of Political Ecology, 27(1), 43–66. 10.2458/v27i1.23335

[R7] Coombes B, Johnson JT, & Howitt R (2014). Indigenous geographies III: Methodological innovation and the unsettling of participatory research. Progress in Human Geography, 38(6), 845–854. 10.1177/0309132513514723

[R8] Corburn J (2007). Community knowledge in environmental health science: Co-producing policy expertise. Environmental Science\ & Policy, 10(2), 150–161. 10.1016/j.envsci.2006.09.004

[R9] Cornish F, Breton N, Moreno-Tabarez U, Delgado J, Rua M, de Graft Aikins A, & Hodgetts D (2023). Participatory action research. Nature Reviews Methods Primers, 3(1). 10.1038/s43586-023-00214-1

[R10] Credo J, & Ingram JC (2021). Perspective developing successful collaborative research partnerships with AI/AN communities. International Journal of Environmental Research and Public Health, 18(17), 9089. 10.3390/ijerph18179089 34501677 PMC8430766

[R11] Curley A (2018). T’áá hwó ají t’éego and the moral economy of Navajo coal workers. Annals of the American Association of Geographers, 109(1), 71–86. 10.1080/24694452.2018.1488576

[R12] Curley A (2021a). Resources is just another word for colonialism. In Himley M, Havice E, & Valdivia G (Eds.), The Routledge handbook of critical resource geography (pp. 79–89). Routledge.

[R13] Curley A (2021b). Livestock, colonialism, and commodity frontiers in the US Southwest. Commodity Frontiers, 3(3), 27–30. 10.18174/cf.2021a18162

[R14] Daigle M (2016). Awawanenitakik: The spatial politics of recognition and relational geographies of indigenous self-determination. Canadian Geographies/Géographies Canadiennes, 60(2), 259–269. 10.1111/cag.12260

[R15] Daigle M (2025). Indigenous peoples’ geographies i: Indigenous spatialities beyond place through relational, mobile and hemispheric & global approaches. Progress in Human Geography, 49(2), 182–193. 10.1177/03091325241283843

[R16] De Leeuw S, Cameron ES, & Greenwood ML (2012). Participatory and community-based research, Indigenous geographies, and the spaces of friendship: A critical engagement. Canadian Geographer, 56(2), 180–194. 10.1111/j.1541-0064.2012.00434.x

[R17] Dionisio MR, Kingham S, Banwell K, & Neville J (2016). Geospatial tools for community engagement in the Christchurch rebuild, New Zealand. Sustainable Cities and Society, 27, 233–243. 10.1016/j.scs.2016.04.007

[R18] Dunn CE (2007). Participatory GIS - a people’s GIS? Progress in Human Geography, 31(5), 616–637. 10.1177/0309132507081493

[R19] Duval-Diop D, Curtis A, & Clark A (2010). Enhancing equity with public participatory GIS in hurricane rebuilding: Faith based organizations, community mapping, and policy advocacy. Community Development, 41(1), 32–49. 10.1080/15575330903288854

[R20] Eichstaedt PH (1994). If you poison us: Uranium and Native Americans. Retrieved October 31, 2021, from https://repository.library.georgetown.edu/handle/10822/869672

[R21] Elwood S, & Leitner H (1998). Gis and community-based planning: Exploring the diversity of neighborhood perspectives and needs. Cartography and Geographic Information Science, 25(2), 77–88. 10.1559/152304098782594553

[R22] Erdei E, Shuey C, Miller C, Hoover J, Cajero M, & Lewis J (2023). Metal mixture exposures and multiplexed autoantibody screening in Navajo communities exposed to uranium mine wastes. Journal of Translational Autoimmunity, 6, 6. 10.1016/j.jtauto.2023.100201 PMC10165442 37169001

[R23] Fegadel AR (2023). Green victimization of native Americans: Uranium mining as a form of toxic colonialism and genocide. Critical Criminology, 31(2), 489–505. 10.1007/s10612-022-09679-0

[R24] Fischer H, Block D, Bosse A, Hawthorne TL, Jung JK, Pearsall H, Rees A, & Shannon J (2022). Doing community geography. Geo Journal, 87(s2), 293–306. 10.1007/s10708-021-10457-8

[R25] Frantz K, & Howitt R (2012). Geography for and with indigenous peoples: Indigenous geographies as challenge and invitation. Geo Journal, 77(6), 727–731. 10.1007/s10708-010-9378-2

[R26] Girlamo C, Lin Y, Hoover J, Beene D, Woldeyohannes T, Liu Z, Campen MJ, MacKenzie D, & Lewis J (2023). Meteorological data source comparison-a case study in geospatial modeling of potential environmental exposure to abandoned uranium mine sites in the Navajo Nation. Environmental Monitoring and Assessment, 195(7). 10.1007/s10661-023-11283-w PMC10258180 37303005

[R27] Haklay M, & Francis L (2017). Participatory GIS and community-based citizen science for environmental justice action. The Routledge Handbook of Environmental Justice, 297–308. 10.4324/9781315678986-24

[R28] Hall K, Crowston K, & Vogel A (2014). How to write a collaboration plan. National Cancer Institute.

[R29] Harney L, McCurry J, Scott J, & Wills J (2016). Developing ``process pragmatism’ to underpin engaged research in human geography. Progress in Human Geography, 40(3), 316–333. 10.1177/0309132515623367

[R30] Hawthorne TL, & Jarrett OS (2018). Developing the next generation of community-based scholars. The Professional Geographer, 70(2), 291–297. 10.1080/00330124.2017.1366780

[R31] Hodge P, & Lester J (2006). Indigenous research: Whose priority? Journeys and possibilities of cross-cultural research in geography. Geographical Research, 44(1), 41–51. 10.1111/j.1745-5871.2006.00370.x

[R32] Hoover JH, Coker ES, Erdei E, Luo L, Begay D, MacKenzie D, Lewis J, & Team NS (2023). Preterm birth and metal mixture exposure among pregnant women from the Navajo Birth Cohort Study. Environmental Health Perspectives, 131(12). 10.1289/EHP10361 PMC10727039 38109118

[R33] Igwe PA, Madichie NO, & Rugara DG (2022). Decolonising research approaches towards non-extractive research. Qualitative Market Research, 25(4), 453–468. 10.1108/QMR-11-2021-0135

[R34] Jung K (2018). Mapping community diversity and e-participation in emergency management: Evidence from WebEOC in the city of San Francisco. International Journal of Emergency Management, 14(3), 275–290. 10.1504/IJEM.2018.094238

[R35] Kater I (2022). Natural and indigenous sciences: Reflections on an attempt to collaborate. Regional Environmental Change, 22(4). 10.1007/s10113-022-01967-3

[R36] Kim M (2018). Project-based community participatory action research using geographic information technologies. Journal of Geography in Higher Education, 42(1), 61–79. 10.1080/03098265.2017.1335294

[R37] King BH (2002). Towards a participatory GIS: Evaluating case studies of participatory rural appraisal and GIS in the developing world. Cartography and Geographic Information Science, 29(1), 43–52. 10.1559/152304002782064565

[R38] Koster R, Baccar K, Lemelin RH, De Leeuw S, Cameron ES, & Greenwood ML (2012). Participatory and community-based research, Indigenous geographies, and the spaces of friendship: A critical engagement. Canadian Geographies / Géographies Canadiennes, 56(2), 180–194. 10.1111/j.1541-0064.2012.00434.x

[R39] Kouritzin S, & Nakagawa S (2018). Toward a non-extractive research ethics for transcultural, translingual research: Perspectives from the coloniser and the colonised. Journal of Multilingual and Multicultural Development, 39(8), 675–687. 10.1080/01434632.2018.1427755

[R40] Kumasaka O, Bronen R, Harrington E, Knox-Hayes J, Laska S, Naquin A, Patrick A, Peterson K, & Tom S (2022). Planning for resettlement: Building partnerships for, by, and with indigenous peoples. Geo Journal, 87(s2), 307–327. 10.1007/s10708-021-10518-y

[R41] Lewis J, Hoover J, & MacKenzie D (2017). Mining and environmental health disparities in Native American communities. Current Environmental Health Reports, 4(2), 130–141. 10.1007/s40572-017-0140-5 28447316 PMC5429369

[R42] Lin Y, Hoover J, Beene D, Erdei E, & Liu Z (2020). Environmental risk mapping of potential abandoned uranium mine contamination on the Navajo Nation, USA, using a GIS-based multi-criteria decision analysis approach. Environmental Science and Pollution Research, 27(24), 30542–30557. 10.1007/s11356-020-09257-3 32468361 PMC7387200

[R43] Lister A, Credo J, & Ingram JC (2024). Determination of uranium in sheep (Ovis aries) liver by inductively coupled plasma mass spectrometry (ICPMS). Journal of Veterinary Medical Research, 11(1), 1263.

[R44] Lister A, Hoover J, & Ingram JC (2023). Determination of uranium in sheep (Ovis aries) kidneys by inductively coupled plasma mass spectrometry (ICPMS). Open Access Journal of Toxicology, 5(4). 10.19080/OAJT.2023.05.555670

[R45] Lister AR (2024). Determination of uranium in sheep kidney, liver, and muscle tissue: The impact of contaminated traditional food sources on the Navajo Nation [Doctoral dissertation]. Northern Arizona University.

[R46] Liu Z, Lin Y, Hoover J, Beene D, Charley PH, & Singer N (2023). Individual level spatial-temporal modelling of exposure potential of livestock in the Cove Wash watershed, Arizona. Annals of GIS, 29(1), 87–107. 10.1080/19475683.2022.2075935 37090684 PMC10117392

[R47] Mamontova N, & Klyachko E (2022). `Process toponymy`: A GIS-based community-engaged approach to Indigenous dynamic place naming systems and vernacular cartography. Cartographica, 57(3), 213–225. 10.3138/cart-2022-0010

[R48] Mares M (2022). Uranium and arsenic accumulation in plants: The impact of abandoned uranium mines on the plant community on the Navajo Nation. last Retrieved March 12, 2025, from https://www.proquest.com/docview/2718099671/abstract/784AF173EAFE4D34PQ/1

[R49] Mason K (2015). Participatory action research: Coproduction, governance and care. Geography Compass, 9(9), 497–507. 10.1111/gec3.12227

[R50] McCall MK, & Minang PA (2005). Assessing participatory GIS for community-based natural resource management: Claiming community forests in Cameroon. The Geographical Journal, 171(4), 340–356. 10.1111/j.1475-4959.2005.00173.x

[R51] Monosky M, & Keeling A (2021). Social considerations in mine closure: Exploring policy and practice in Nunavik, Quebec. The Northern Review, 52(52), 29–61. 10.22584/nr52.2021.002

[R52] Nelson S, & McGregor D (2014). Decolonizing the discipline? Questions and methods in indigenous geography. Canadian Journal of Native Education, 37(1), 105–121.

[R53] Nez TR (2020). Cove mutton consumption survey: Policy insights to address uranium contamination of Navajo traditional mutton (Order No. 28256663) [Dissertations & Theses @ Northern Arizona University; ProQuest Dissertations & Theses Global. (2471743232)]. http://search.proquest.com.libproxy.nau.edu/dissertations-theses/cove-muttonconsumption-survey-policy-insights/docview/2471743232/se-2

[R54] Pasternak J (2010). Yellow dirt: An American story of a poisoned land and a people betrayed. Simon and Schuster.

[R55] Radcliffe SA (2022). Decolonizing geography: An introduction. John Wiley & Sons.

[R56] Radil SM, & Anderson MB (2019). Rethinking PGIS: Participatory or (post)political GIS? Progress in Human Geography, 43(2), 195–213. 10.1177/0309132517750774

[R57] Roberts D (2012). Agency for Toxic Substances and Disease Registry case studies in environmental medicine (CSEM) uranium toxicity. www.atsdr.cdc.gov/csem/

[R58] Robinson JA (2010). Syracuse community geography: Evaluating a new approach to public participation geographic information systems [Doctoral thesis]. University of North Carolina at Chapel Hill. https://cdr.lib.unc.edu/concern/dissertations/6682x5304

[R59] Rock T, Camplain R, Teufel-Shone NI, & Ingram JC (2019). Traditional sheep consumption by Navajo people in Cameron, Arizona. International Journal of Environmental Research and Public Health, 16(21), 4195. 10.3390/ijerph16214195 31671510 PMC6862166

[R60] Rock T, & Ingram JC (2020). Traditional ecological knowledge policy considerations for abandoned uranium mines on Navajo Nation. Human Biology, 92(1), 19–26. 10.13110/humanbiology.92.1.01 33231023 PMC8477793

[R61] Rundstrom RA (1995). Gis, Indigenous peoples, and epistemological diversity. Cartography and Geographic Information Systems, 22(1), 45–57. 10.1559/152304095782540564

[R62] Samuel-Nakamura C (2020). Using traditional methods for collaborative fieldwork in a uranium food chain study on Dine lands in the US Southwest. Sustainability, 12(17), 6886. 10.3390/su12176886

[R63] Shannon J, Hankins KB, Shelton T, Bosse AJ, Scott D, Block D, Fischer H, Eaves LTE, Jung JK, Robinson J, Solís P, Pearsall H, Rees A, & Nicolas A (2021). Community geography: Toward a disciplinary framework. Progress in Human Geography, 45(5), 1147–1168. 10.1177/0309132520961468

[R64] Sultana F (2014). Gendering climate change: Geographical insights. The Professional Geographer, 66(3), 372–381. 10.1080/00330124.2013.821730

[R65] Tobias JK, Richmond CAM, & Luginaah I (2013). Community-based participatory research (CBPR) with indigenous communities: Producing respectful and reciprocal research. Journal of Empirical Research on Human Research Ethics, 8(2), 129–140. 10.1525/jer.2013.8.2.129 23651937

[R66] U.S. Environmental Protection Agency. (2024). EPA adds sites to the Superfund National Priorities List, including the Lukachukai Mountains Mining District in Navajo Nation. Retrieved May 12, 2025, from https://www.epa.gov/newsreleases/epa-adds-sites-superfund-national-priorities-list-including-lukachukai-mountains

[R67] Voyles TB (2015). Wastelanding: Legacies of uranium mining in Navajo country. University of Minnesota Press.

[R68] Zurba M, Maclean K, Woodward E, & Islam D (2019). Amplifying indigenous community participation in place-based research through boundary work. Progress in Human Geography, 43(6), 1020–1043. 10.1177/0309132518807758

